# Identification of 5 novel genes methylated in breast and other epithelial cancers

**DOI:** 10.1186/1476-4598-9-51

**Published:** 2010-03-05

**Authors:** Victoria K Hill, Luke B Hesson, Temuujin Dansranjavin, Ashraf Dallol, Ivan Bieche, Sophie Vacher, Stella Tommasi, Timothy Dobbins, Dean Gentle, David Euhus, Cheryl Lewis, Reinhard Dammann, Robyn L Ward, John Minna, Eammon R Maher, Gerd P Pfeifer, Farida Latif

**Affiliations:** 1 Department of Medical and Molecular Genetics, School of Clinical and Experimental Medicine, College of Medical and Dental Sciences, University of Birmingham, Birmingham B15 2TT, UK; 2 Adult Cancer Program, Lowy Cancer Research Centre and Prince of Wales Clinical School, Faculty of Medicine, University of New South Wales, NSW2052, Australia; 3 Institute for Genetics, Justus Liebig University Giessen, Heinrich-Buff-Ring 58-62, D-35392 Giessen, Germany; 4 Oncogenetic Laboratory, INSERM U 735, Centre René Huguenin, Saint Cloud, France; 5 Beckman Research Institute, City of Hope, 1500 E Duarte Road, Duarte, CA 91010. USA; 6 Department of Surgery, University of Texas Southwestern Medical Center, Dallas, Texas, USA; 7 Hamon Center for Therapeutic Oncology Research, The University of Texas Southwestern Medical Center at Dallas, Dallas, Texas, USA

## Abstract

**Background:**

There are several high throughput approaches to identify methylated genes in cancer.  We utilized one such recently developed approach, MIRA (methylated-CpG island recovery assay) combined with CpG island arrays to identify novel genes that are epigenetically inactivated in breast cancer.

**Results:**

Using this approach we identified numerous CpG islands that demonstrated aberrant DNA methylation in breast cancer cell lines. Using a combination of COBRA and sequencing of bisulphite modified DNA, we confirmed 5 novel genes frequently methylated in breast tumours; *EMILIN2, SALL1*, *DBC1*, *FBLN2 *and *CIDE-A*. Methylation frequencies ranged from between 25% and 63% in primary breast tumours, whilst matched normal breast tissue DNA was either unmethylated or demonstrated a much lower frequency of methylation compared to malignant breast tissue DNA. Furthermore expression of the above 5 genes was shown to be restored following treatment with a demethylating agent in methylated breast cancer cell lines. We have expanded this analysis across three other common epithelial cancers (lung, colorectal, prostate). We demonstrate that the above genes show varying levels of methylation in these cancers. Lastly and most importantly methylation of *EMILIN2 *was associated with poorer clinical outcome in breast cancer and was strongly associated with estrogen receptor as well as progesterone receptor positive breast cancers.

**Conclusion:**

The combination of the MIRA assay with CpG island arrays is a very useful technique for identifying epigenetically inactivated genes in cancer genomes and can provide molecular markers for early cancer diagnosis, prognosis and epigenetic therapy.

## Background

In most Western countries breast cancer is a leading cause of cancer related deaths in women. Breast cancer accounts for over one million of the estimated 10 million cancers that are diagnosed globally each year in both males and females and claimed about 375,000 deaths in the year 2000 [1]. The use of screening mammography and MRI has led to a decline in breast cancer-related mortality. But these screening procedures can also lead to overdiagnosis and false positive cases leading to unnecessary treatment. Therefore there is a critical need to develop molecular biomarkers that can detect early stage disease so that treatment can begin promptly.

Genetic as well as epigenetic changes play crucial roles in tumour development and progression. Alterations in DNA methylation include both genome-wide hypomethylation and hypermethylation events. Promoter hypermethylation at specific gene loci is a major mechanism of epigenetic inactivation in cancer cells leading to gene silencing. A recent genome-wide study in breast and colon cancer led to identification of tumour suppressor genes that are inactivated by both genetic and epigenetic events and importantly reduced expression of a subset of these genes correlated with poor clinical outcome [2]. Although the list of hypermethylated genes in breast cancer is growing, only a few show promise as biomarkers for early detection and risk assessment [3,4]. We and others identified *RASSF1A *as one of the most highly methylated genes in many cancer types including breast cancer [5,6]. Identification of more genes of this type would facilitate the development of sensitive molecular markers across multiple malignancies.

In the present study we employed a genome-wide approach to identify methylated genes in breast cancer and further investigated these genes in other common epithelial cancers. For the high throughput genome-wide DNA methylation approach we utilized a very sensitive assay recently developed in our laboratory (Gerd Pfeifer lab). The methylated-CpG island recovery assay (MIRA) is based on the high affinity of the MBD2/MBD3L1 complex for methylated DNA and allows one to detect cell type-dependent differences in DNA methylation when used in combination with CpG island arrays [7,8].

## Results

### Genome-wide approach identifies methylated genes in breast cancer

**Figure 1:**
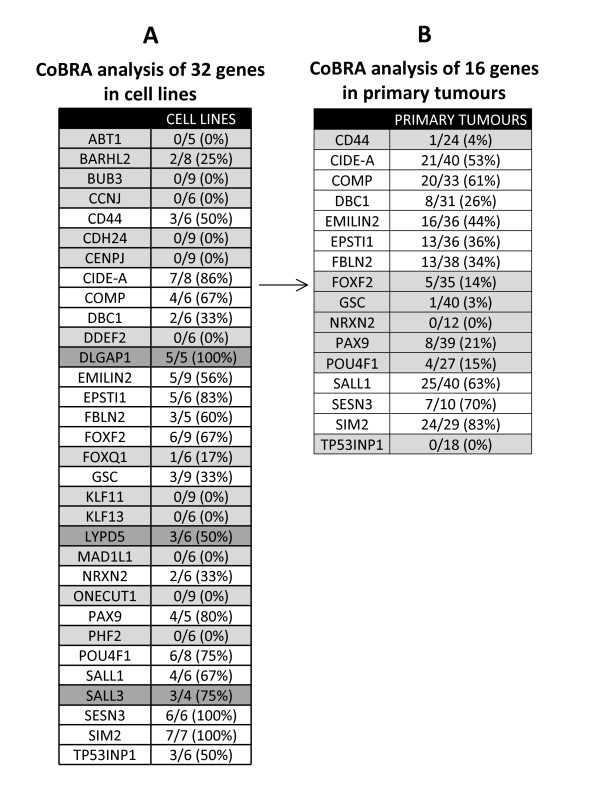
**Stepwise analysis of 32 selected genes in breast cancer**. (A) Cell line analysis confirmed frequent methylation (>30% of samples) in cancer cell lines but not normal/control breast tissue in 16 genes (highlighted in white), which were selected for further analysis. Thirteen genes (highlighted in light grey) did not show methylation in the region analysed by our CoBRA primers and were removed from further analysis. Three genes (highlighted in dark grey) showed frequent methylation in cancer cell lines but also normal breast tissue and were removed from further analysis. (B) Analysis of the 16 genes found to be frequently methylated in cell lines were analysed in primary breast cancer patient samples. Frequent methylation (>25% of samples) was observed in nine of these genes (highlighted in white) and were carried forward for expression analysis.

We used the MIRA assay in combination with genome-wide CpG island arrays containing 27,800 loci to identify methylated CpG islands in 5 breast cancer cell lines (HCC1806, HCC1419, HCC1143, HCC1937, HCC1395) relative to normal human mammary epithelial cells (NHMEC) [7,8]. A primary selection based on probes showing > 5 fold enrichment of the methylated fraction in breast cancer cell lines versus NHMEC cell line DNA across 3 or more cell lines or a more stringent selection with probes scoring greater than 10 in 4 or more cell lines yielded a large number of loci showing methylation across breast cancer cell lines (see additional 1). These included genes/loci previously reported to be methylated in breast cancer (for example *SIM1*, *PAX6*, *DLX4*, *RUNX3*). In order to reduce the list to manageable numbers we carried out bioinformatic analyses to gather information on the expression of target genes in breast tissue, confirmation of the presence of a CpG island within the likely promoter region of target genes and functional annotation to determine likely roles in tumourigenesis, see materials and methods for more information. This led to a selection of 32 novel genes for further analysis (Figure 1, see additional file 2) in a stepwise fashion including the following stages:

1. Methylation analysis in breast cancer cell lines and normal control breast tissue DNA.

2. Methylation analysis in a panel of breast tumours (n = 40)

3. Gene expression analysis before and after 5-aza-dC treatment

4. Methylation analysis in an independent cohort of paired normal/tumour breast DNA panel (n = 20)

5. Expand methylation analysis to include lung, colorectal and prostate cancer, for the genes showing tumour-specific methylation in breast cancer.

### Methylation analysis in breast tumours and breast tumour cell lines

**Figure 2:**
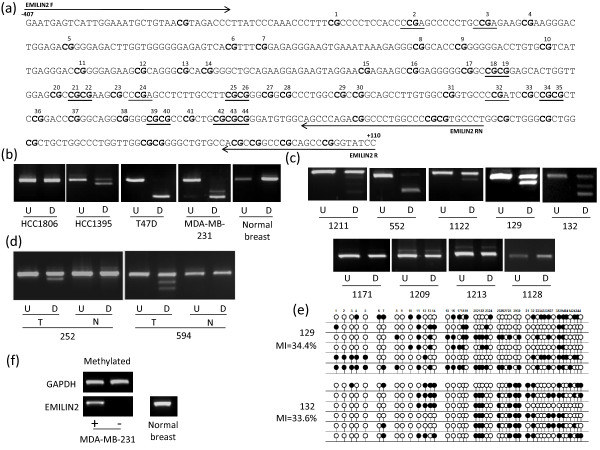
**Methylation analysis of *EMILIN2***. (a) The region analysed by CoBRA and sequencing is shown with CpG dinucleotides numbered and shown in bold and BstUI and TaqαI restriction sites underlined. The position of CoBRA primers is also shown, covering the region between -407 bp and +110 bp with respect to the transcription start site. (b) CoBRA results are shown with undigested (U) PCR product next to digested (D) PCR product for breast cancer cell lines and normal breast. (c) CoBRA results for primary breast tumours. (d) CoBRA results for breast tumour normal pairs. (e) Selected PCR products were cloned and sequenced. Individual alleles of each of the two samples shown are represented by a line, black and white circles represent methylated and unmethylated CpG dinucleotides respectively. The frequency of methylation within the amplified region for each sample is indicated by the methylation index (MI). (f) *EMILIN2 *expression in normal breast tissue and re-expression in methylated breast cancer cell line MDA-MB-231 following the addition of 5-aza-2'deoxycitidine is shown.

**Figure 3:**
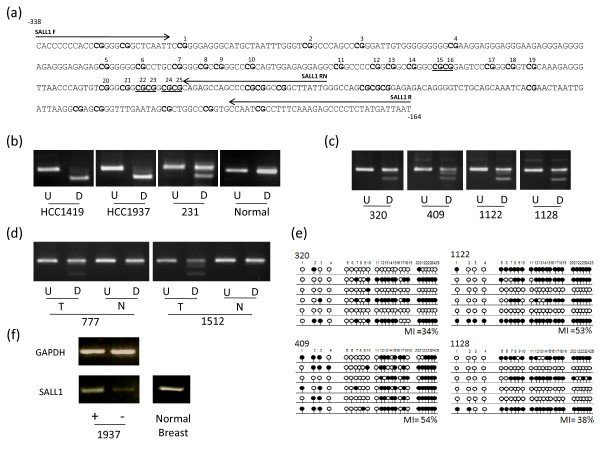
**Methylation analysis of *SALL1***. (a) The region analysed by CoBRA and sequencing is shown with CpG dinucleotides numbered and shown in bold and BstUI restriction sites underlined. The position of CoBRA primers is also shown, covering the region between -338 bp and -164 bp with respect to the transcription start site. (b) CoBRA results are shown with undigested (U) PCR product next to digested (D) PCR product for breast cancer cell lines and normal breast. (c) CoBRA results for primary breast tumours. (d) CoBRA results for breast tumour normal pairs. (e) Selected PCR products were cloned and sequenced. Individual alleles of each of the four samples shown are represented by a line, black and white circles represent methylated and unmethylated CpG dinucleotides respectively. The frequency of methylation within the amplified region for each sample is indicated by the methylation index (MI). (f) *SALL1 *expression in normal breast tissue and upregulation in methylated breast cancer cell line HCC1937 following the addition of 5-aza-2'deoxycitidine is shown.

**Figure 4:**
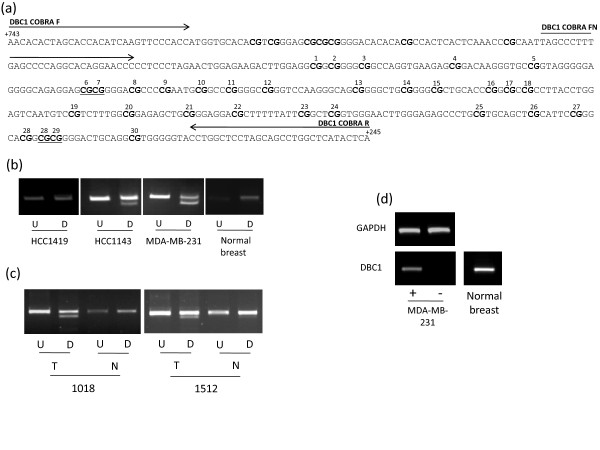
**Methylation analysis of *DBC1***. (a) The region analysed by CoBRA and sequencing is shown with CpG dinucleotides numbered and shown in bold and BstUI restriction sites underlined. The position of CoBRA primers is also shown, covering the region between +743 and +245 with respect to the transcription start site. (b) CoBRA results are shown with undigested (U) PCR product next to digested (D) PCR product for breast cancer cell lines and normal breast. (c) CoBRA results for breast tumour normal pairs. (d) *DBC1 *expression in normal breast tissue and re-expression in methylated breast cancer cell line MDA-MB-231 following the addition of 5-aza-2'deoxycitidine is shown.

**Figure 5:**
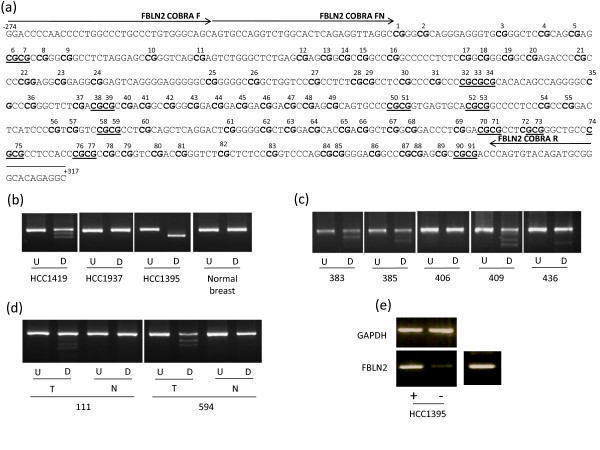
**Methylation analysis of *FBLN2***. (a) The region analysed by CoBRA and sequencing is shown with CpG dinucleotides numbered and shown in bold and BstUI restriction sites underlined. The position of CoBRA primers is also shown, covering the region between -274 and +317 with respect to the transcription start site. (b) CoBRA results are shown with undigested (U) PCR product next to digested (D) PCR product for breast cancer cell lines and normal breast. (c) CoBRA results for primary breast tumours. (d) CoBRA results for breast tumour normal pairs. (e) *FBLN2 *expression in normal breast tissue and upregulation in methylated breast cancer cell line HCC1395 following the addition of 5-aza-2'deoxycitidine is shown.

**Figure 6:**
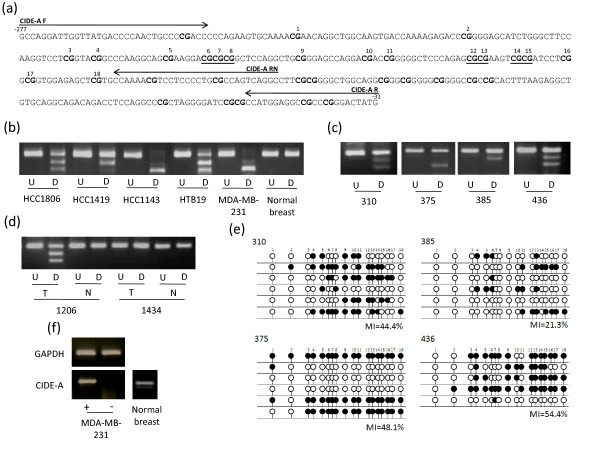
**Methylation analysis of *CIDE-A***. (a) The region analysed by CoBRA and sequencing is shown with CpG dinucleotides numbered and shown in bold and BstUI restriction sites underlined. The positions of CoBRA primers are also shown, covering the region between -277 and -31 with respect to the transcription start site. (b) CoBRA results are shown with undigested (U) PCR product next to digested (D) PCR product for breast cancer cell lines and normal breast. (c) CoBRA results for primary breast tumours. (d) CoBRA results for breast tumour normal pairs. (e) Selected PCR products were cloned and sequenced. Individual alleles of each of the four samples shown are represented by a line, black and white circles represent methylated and unmethylated CpG dinucleotides respectively. The frequency of methylation within the amplified region for each sample is indicated by the methylation index (MI). (f) *CIDE-A *expression in normal breast tissue and re-expression in methylated breast cancer cell line MBA-MD-231 following the addition of 5-aza-2'deoxycitidine is shown.

We used COBRA digest to ascertain the methylation levels across a panel of 9 breast cancer cell lines (including the 5 lines used for MIRA) in CpG island regions upstream of the transcription start site. We confirmed frequent methylation (>30%) for 16 loci in breast cancer cell lines. These loci were unmethylated in DNA from normal/control breast. Sixteen loci either showed low or no methylation in the panel of breast cancer cell lines or were equally methylated in normal control breast DNA in the region analysed by our COBRA primers and were removed from further analysis (Figure 1). The remaining 16 positive genes demonstrating frequent methylation in breast cancer cell lines were analysed further by COBRA digest in a panel of primary breast tumours (n = 40). Nine (CIDE-A, COMP, DBC1, EMILIN2, EPSTI1, FBLN2, SALL1, SESN3, SIM2) of these genes (56%) demonstrated frequent methylation in primary breast tumours (frequency ranging from 26-63%) (Figure 1, 2, 3, 4, 5, 6)

### Expression analysis in breast tumour cell lines and normal breast

The above nine frequently methylated genes were shown to be expressed in normal breast tissue (Figure 2, 3, 4, 5, 6). In order to determine the role of methylation in gene silencing, we treated breast cancer cell lines with the demethylating agent 5-aza-dC. Eight (*CIDE-A*, *COMP*, *DBC1*, *EMILIN2*, *EPSTI1*, *FBLN2*, *SALL1*, *SIM2*) of the above nine genes showed re-expression after treatment of the methylated cell lines with 5aza-dC (Figure 2, 3, 4, 5, 6). Hence methylation suppressed gene expression, whilst *SESN3 *showed no change in expression before and after treatment in both methylated and unmethylated cell lines. *SESN3 *was therefore not considered for further analysis.

### Methylation analysis in paired normal/tumour breast cases

To determine if methylation of the eight remaining genes was cancer-specific (i.e. tumour acquired), we analysed an independent set of paired normal/tumour breast DNA samples (n = 20). Five genes (62.5%) demonstrated tumour acquired methylation (*EMILIN2*, *SALL1*, *DBC1*, *FBLN2 *and *CIDE-A*) (Table 1, Figures 2, 3, 4, 5, 6), whilst the remaining 3 (*COMP*, *EPSTI1*, *SIM2*) showed equal methylation in the corresponding normal breast tissue DNA. The COBRA results for these 5 genes were confirmed by cloning and sequencing of bisulphite modified DNA in primary breast tumours (Figures 2, 3, 4, 5, 6). In majority of the breast tumour samples we also detected the unmethylated product. This is likely to be due to the contamination from normal cells, since none of the tumours were microdissected and due to the heterogeneous nature of the primary tumour samples.

**Table 1:** Methylation frequencies in breast cancer

	CIDE-A	DBC1	EMILIN2	FBLN2	SALL1
Cell lines	7/8 (88%)	2/6 (34%)	5/9 (56%)	3/5 (60%)	4/6 (67%)
Tumours	21/40 (53%)	8/31 (26%)	16/36 (45%)	13/38 (34%)	25/40 (63%)
Matched pairs	7/15 (47%)	4/16 (25%)	4/10 (40%)	6/19 (32%)	10/20 (50%)*

Frequencies of methylation in breast cancer cell lines, primary breast tumours and matched breast tumour/normal pair samples are shown for *EMILIN2*, *SALL1*, *DBC1*, *FBLN2 *and *CIDE-A*. All tumour/normal pair figures represent the number of pairs that show tumour specific methylation, (*) indicates that one of these pairs also showed methylation in the corresponding normal.

### Methylation analysis in other epithelial cancers

To explore whether the above 5 genes showing tumour-specific methylation in breast cancer were methylated in other epithelial cancers besides breast, we analysed a panel of cancer cell lines corresponding to three common epithelial cancers (lung, colorectal and prostate). Lung tumour cell lines (NSCLC) demonstrated frequent methylation of *CIDE-A*, *EMILN2*, *FBLN2 *and *SALL1*. *DBC1 *methylation in lung cancer has been reported previously hence it was not analysed in this study [9]. Colorectal tumour lines showed frequent methylation of all 5 genes, whilst prostate tumour lines demonstrated frequent methylation of *CIDE-A*, *DBC1*, *EMILIN2*, *SALL1 *and less frequent methylation for *FBLN2 *(Table 2).

**Table 2:** Methylation frequencies in lung, colorectal and prostate cancers.

	CIDE-A	DBC1	EMILIN2	FBLN2	SALL1
Lung tumour lines	11/15	not done	4/10	7/9	4/13
Lung tumours	1/16	not done	1/13	0/11	4/16
Lung benign	0/16	not done	1/17	0/10	3/17
					
Colorectal tumour lines	8/8	8/8	4/5	4/4	6/6
Colorectal tumours	7/8	8/8	19/57	54/58	44/53
Colorectal adenomas	7/7	8/8	7/22	23/23	17/19
Colorectal benign	16/16	16/16	1/82	74/80	30/79
					
Prostate tumour lines	3/4	2/5	3/5	1/5	3/4
Prostate tumours	3/14	1/14*	0/14	5/14	5/14
Prostate benign	1/4	2/4*	0/4	0/4	1/4

Methylation frequencies are shown for lung, colorectal and prostate cancer cell lines and primary tumours and normal tissue. (*) very weak methylation. *DBC1 *methylation in lung cancer has been demonstrated previously, hence in this study *DBC1 *was not analysed in lung cancer.

### To demonstrate tumour specific methylation in other epithelial cancers

The above genes were further analyzed in primary tumours for the tumour types that demonstrated frequent methylation in the corresponding tumour cell lines. Where applicable N/T paired DNA was analysed (Table 2).

**Figure 7:**
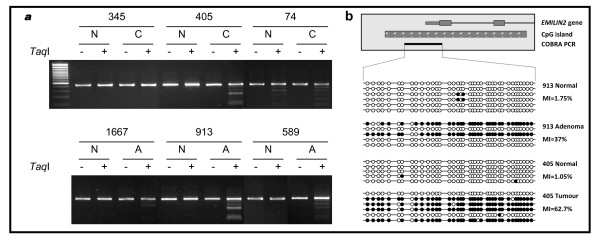
**Methylation analysis of *EMILIN2 *in colorectal cancer**. (a) COBRA results are shown with undigested (-) PCR product next to digested (+) PCR product for matching normal (N) and colorectal carcinoma (C) in the upper panel and matching normal (N) and adenoma (A) in the lower panel. (b) Selected PCR products were cloned and sequenced. Individual alleles of each of the four samples shown are represented by a line, black and white circles represent methylated and unmethylated CpG dinucleotides respectively. The frequency of methylation within the amplified region for each sample is indicated by the methylation index (MI).

*EMILIN2 *was methylated in 33% colorectal carcinomas (CRC) and in 32% adenomas, whilst only 1.2% of corresponding normal tissues demonstrated any methylation. Methylation was confirmed using cloning and sequencing of bisulphite modified DNA (Figure 7). Whilst NSCLC and prostate tumours were either unmethylated or showed very low level frequency of methylation in both tumours and corresponding normals (Table 2).

*SALL1 *was methylated in 83% of CRC and in 89% of adenomas, whilst methylation frequency in corresponding normals was much less (38%). *SALL1 *demonstrated similar frequencies of methylation in tumour and matched normal samples for both NSCLC and prostate tumours (Table 2).

*DBC1 *was frequently methylated in CRC and prostate cancer cell lines (we did not analyse NSCLC since *DBC1 *is already known to be methylated in this cancer). In CRC and adenomas the methylation frequency was 100% but it was not cancer specific, all corresponding normals were also methylated. This could indicate that *DBC1 *undergoes tissue-specific methylation in certain tissues but not in others. Whilst weak methylation was seen in 1 out of 14 prostate tumours and 2 out of 4 normals (Table 2).

*FBLN2 *demonstrated tumour specific methylation in prostate tumours (36%) and was unmethylated in lung tumours. *FBLN2 *similar to *DBC1 *demonstrated very high frequency of methylation in colorectal tumours, adenomas and corresponding normal samples (Table 2).

*CIDE-A *was found to be methylated in 7/8 analysed primary CRC tumours and in all adenomas and corresponding normals. It has been recently demonstrated that *CIDE-A *expression is epigenetically controlled in certain tissue and cell types [10], which when taken in consideration with our methylation analysis, suggests that normal colorectal tissue does not express *CIDE-A*. Whilst a similar percentage of methylation frequency was seen in prostate tumours and corresponding normals (21% and 25% respectively). *CIDE-A *was methylated in 1/16 NSCLC and 0/16 matched normals (Table 2).

### Methylation status and clinicopathological parameters

**Figure 8:**
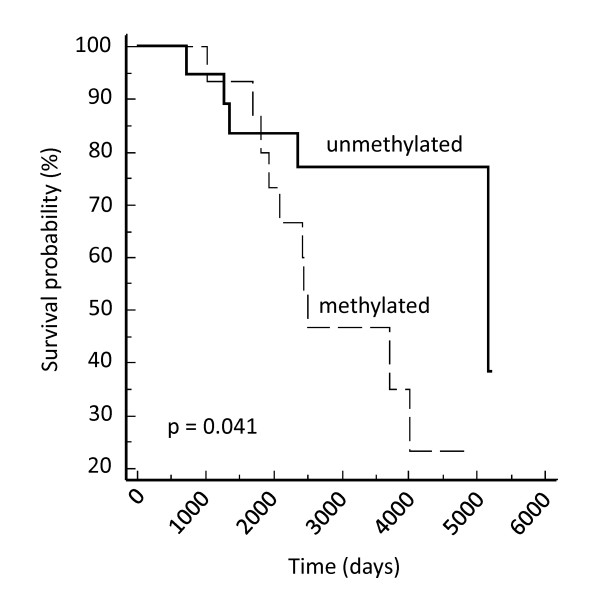
***EMILIN2 *survival analysis in breast tumours**. Kaplan Meier survival analysis for *EMILIN2 *methylation status and relapse free survival (P = 0.041).

The most interesting finding in the breast cancer cohort was that the methylation of *EMILIN2 *was associated with relapse (P = 0.031), presence of lymph node metastases (P = 0.047), with ER and PR positive tumours (P = 0.0009; 0.0082 respectively) and poor relapse free survival (P = 0.041) (Figure 8). *CIDE-A *methylation was associated with ER positive tumours (P = 0.016), whilst *FBLN2 *methylation was associated with PR positive tumours (P = 0.013).

### Expression analysis of *EMILIN2 *in breast tumours

**Figure 9:**
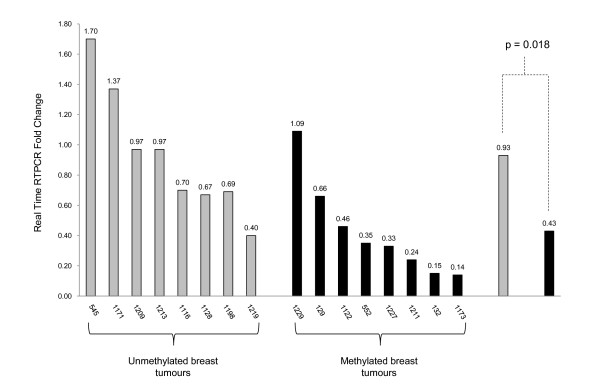
***EMILIN2 *expression analysis in breast tumours**. *EMILIN2 *mRNA expression is downregulated by promoter region hypermethylation in breast tumours. *EMILIN2 *expression was detected by quantitative real-time RT-PCR in *EMILIN2 *unmethylated and methylated breast tumours (Figure 2c shows the methylation status for some of these tumours). The averages for both unmethylated and methylated samples are also shown and a t-test has been used to determine the statistical significance between the two (p = 0.018).

*EMILIN2 *appears to be the most interesting gene due to the association between its methylation status and clinical-pathological features including survival. To confirm that the methylation of *EMILIN2 *is functionally relevant, we determined *EMILIN2 *expression in a subset of primary breast tumours (good quality RNA was available for these samples) using quantitative real-time RT-PCR. Expression analysis revealed that the breast tumours with *EMILIN2 *methylation showed lower levels of *EMILIN2 *expression compared to unmethylated tumours (Figure 9).

## Discussion

Using a high throughput genome-wide approach we have identified 5 genes showing frequent tumour acquired methylation in breast cancer. To the best of our knowledge methylation of *EMILIN2*, *SALL1*, *DBC1*, *FBLN2*, or *CIDE-A *has not been reported previously in breast cancer. Furthermore we demonstrate that the methylation is cancer specific and leads to gene silencing. In addition we found that *EMILIN2*, *FBLN2 *and *SALL1 *were also frequently methylated in other common epithelial cancers, *EMILIN2 *and *SALL1 *in colorectal cancer and *FBLN2 *in prostate cancer. To the best of our knowledge *EMILIN2 *and *CIDE-A *methylation in primary tumours has not been previously reported, *SALL1 *and *FBLN2 *methylation has been previously reported in acute lymphocytic leukemia [11,12] and *DBC1 *methylation has been reported in NSCLC, bladder cancer, oral cancer and in leukaemia [9,13-15].

EMILIN2 belongs to a family of extracellular matrix (ECM) glycoproteins containing an EMI domain at the N-terminus and a gC1q domain at the C-terminus. EMILIN2 has been shown to suppress growth of cancer cells [16] and triggers apoptosis in cancer cells via the extrinsic apoptosis pathway following EMILIN2 binding to the trail receptors DR4 and DR5.

CIDE-A belongs to a novel family of cell death inducing DFF45 (DNA fragmentation factor-45) like effector proteins. In humans there are three family members (CIDE-A, CIDE-B, CIDE-C). In cell lines overexpression of CIDE-A leads to caspase-independent cell death associated with DNA fragmentation [17]. In addition, mice lacking functional Cidea have higher metablic rates and lipolysis in the brown adipose tissue as well as being resistant to diet-induced obesity and diabetes as compared to their wildtype littermates [18]. A recent report indicates that CpG methylation plays a critical role in determining tissue and cell specific expression of the CIDE-A gene [10].

FBLN2 encodes for an extracelluar matrix protein, belonging to a 6 member family of fibulins. FBLN2 binds various extracellular ligands and calcium. It acts as a scaffold protein in the ECM by binding to a variety of ligands including type IV collagen, fibronectin, fibrinogen, fibrillin, laminins, aggrecan and versican. It was recently shown to be downregulated in breast cancer cell lines and primary breast tumours. Reintroduction of FBLN2 into breast cancer cell lines lacking FBLN2 reduced cell motility and invasion in vitro but had no effect on cell growth and adhesion. Whilst, loss of FBLN2 expression increased cell migration and invasion [19]. Recently another member of the Fibulin gene family, *FBLN3 *(*EFEMP1*) was shown to be frequently methylated in breast tumours [20].

DBC1 (deleted in bladder cancer 1) was identified from a common region of loss of heterozygosity at 9q32-q33 in bladder cancer. DBC1 has been shown to be frequently methylated in bladder cancer, NSCLC, oral cancer and acute lymphocytic leukemia [9,13-15]. Exogenous expression of DBC1 in NSCLC and in bladder cancer cell lines lacking DBC1 expression inhibited cell growth [9,21]. It has also been demonstrated that DBC1 plays a role in cell cycle control [21].

SALL1 is a human homologue of the Drosophila region-specific homeotic gene spalt (sal). The SALL1 gene product is a zinc finger protein thought to act as a transcription factor and maybe part of the NuRD histone deacetylase complex. Mice lacking Sall1 die in the perinatal period from kidney agenesis [22]. Townes-Brocks syndrome affecting limb, ear, kidney and heart development is caused by defects in the SALL1 gene [23]. SALL1 was recently demonstrated to be hypermethylated in acute lymphocytic leukemia [11].

It has been demonstrated that methylated genes often reside in regions of chromosomal loss [2]. *SALL1 *resides at 16q12.1- a region demonstrated to undergo loss of heterozygosity (LOH) in breast, prostate, ovarian cancer and in retinoblastoma [24,25]. *EMILIN2 *is located at 18p11.3 in a region showing LOH in NSCLC, CRC and breast cancer [26]. *FBLN2 *is located at 3p25.1 in a region well documented for allelic loss in renal cell carcinoma and breast cancer [27,28]. *CIDE-A *is located at 18p11.2 in a region showing allelic loss in esophageal squamous cell carcinoma [29] and DBC1 was identified from a region of frequent loss of hetrozygosity at 9q32-33 in bladder cancer [30].

Breast cancer is a hormone dependent cancer. In patients with breast cancer estrogen receptor (ER) status is an important treatment and prognostic factor. The response of breast cancer patients to endocrine therapy is guided by the expression of estrogen and/or progesterone receptors. Breast cancers that are positive for estrogen receptor and/or progesterone receptor respond better to endocrine therapy compared to receptor negative breast cancers. Our findings indicate that methylation of *EMILIN2*, *CIDE-A *and *FBLN2 *are associated with positive estrogen and or progesterone receptor status, this may help in further refining breast cancers that are more likely to benefit from endocrine therapy.

In our cohort of breast cancer cases, *EMILIN2 *methylation also correlated with lymph node metastases, relapse and poor survival, hence *EMILIN2 *methylation is associated with less favourable prognosis. It will be of interest to analyse larger sample numbers to determine if *EMILIN2 *methylation status can be utilised as a prognostic marker in breast cancer.

## Conclusion

In summary we have identified 5 (*EMILIN2*, *SALL1*, *DBC1*, *FBLN2*, *CIDE-A*) genes that demonstrated frequent tumour acquired methylation in breast cancer. In addition, *EMILIN2 *and *SALL1 *also show frequent methylation in colorectal cancer and *FBLN2 *shows frequent tumour-specific methylation in prostate cancer. Identification of these methylated genes will help in elucidating the pathology of the affected cancers and in developing epigenetic markers across epithelial and other cancers.

## Methods

### Cancer cell lines and patient samples

Five breast cancer cell lines (HCC1806, HCC1419, HCC1395, HCC1143 and HCC1937) and two normal human mammary epithelial cell lines (NHMEC lines 1585T and 3736T) were used for MIRA. CoBRA analysis used an additional four breast cancer cell lines (MCF7, T47D, MDA-MB-231 and HTB19) and one normal breast tissue DNA sample (AMS Biotechnology), replacing the NHMEC lines. Cell lines were grown as described previously [31,32]. Clinical and pathological features of the breast cancer cell lines are described in reference 31 and additional file 3. A set of 40 breast tumours (ductal carcinomas) and an independent set of further 20 breast tumours (ductal carcinomas) with corresponding normal breast tissue DNA were analysed. Additional file 4 gives the clinical-pathological information for the breast tumours used in this study. Ethical guidelines were followed for sample collection. A cell line panel consisting of nine colorectal, fifteen lung and five prostate cancer cell lines was used for initial analyses in these cancers. Further work was then carried out using colorectal adenomas and tumours, non small cell lung carcinomas and prostate tumours together with corresponding non cancerous tissue.

### MIRA Assay

The MIRA assay was carried out on five breast cancer cell lines (HCC1806, HCC1419, HCC1395, HCC1143 and HCC1937) and two normal mammary epithelial cell lines (NHMEC lines 1585T and 3736T). The MIRA assay and chip hybridisation procedure was carried out as described previously in references 7 and 8. Human CpG island microarrays containing 27800 CpG islands covering 21 MB were purchased from Agilent Technologies.

### Candidate Selection

Methylation enrichment factors of tumour to normal were calculated for all probes. This data was then analysed as follows:

• First selection, a single probe with a greater than 5-fold increase in MIRA-enriched fraction of tumour versus normal in 3 or more cell lines. This selected for 14,875 probes representing 6,912 individual hits.

• From these hits were removed any that did not represent a known, named gene. This selection removed 145 Open reading frames (Orfs), 3225 Chromosomal regions, 29 miRNAs, 143 Predicted genes (LOCs, MGCs, FLJs and KIAs) and 23 Agilent Plate related hits. This selection retained 3,347 genes (10,840 probes) which represent a low stringency candidate list.

• The low stringency candidate probe list was further refined by the removal of all probes not designated to be within promoter or divergent promoter regions. This selection retained 3,352 probes representing 1268 genes.

• Finally, a stricter selection criteria of a single probe with a greater than 10-fold increase in 4 or more cell lines was applied. This selection retained 895 probes representing 276 genes which represent a high stringency candidate list (see additional file 1)

A large number of target genes were identified using both approaches, particularly the low stringency approach. The low stringency candidate list was compared to a publically available expression array data for an experiment demonstrating the re-expression of genes in the MCF7 breast cancer cell line after treatment with a de-methylating agent (GEO data base accession ID GSE5816). Genes in both the low stringency candidate list and the re-expressed list were then considered. Functional annotation of target genes from both lists was carried out using the functional annotation table function in DAVID http://david.abcc.ncifcrf.gov[33,34]. Additional file 2 shows the results from this analysis for the 32 target genes chosen to be analysed in this study. We also carried out literature searches using Pubmed http://www.ncbi.nlm.nih.gov/pubmed/ for the keywords (breast cancer, Cancer & Methylation) and searched SAGE expression data(GeneCards-http://www.genecards.org/cgi-bin/cardsearch) and Oncomine https://www.oncomine.org/. UCSC genome browser http://genome.ucsc.edu/ was used to confirm the presence of CpG islands within the likely promoter regions of genes.

### Methylation analysis

Combined bisulphite restriction analysis (CoBRA) was used to analyse all genes in this study. Bisulphite modification of DNA was carried out as described previously [35]. Semi-nested CoBRA primers were designed to amplify specific regions within the CpG island close to the start of transcription for all 32 genes (primer sequences are shown in additional file 5 for the final 5 positive genes). Digestion of PCR products with BstUI (CGCG) or TaqαI (TCGA) was carried out overnight at 37°C or 65°C respectively prior to visualisation on a 2% agarose gel. *In vitro *methylated DNA was used as a positive control. Confirmation of CoBRA results was obtained by bisulphite sequencing. PCR products from selected samples were cloned into the pGEM-T easy vector (Promega) according to manufacturers' instructions. Single colony PCR was carried out on up to 12 colonies for each cloned sample and methylation index (MI) for each sample calculated as a percentage; number of CpG dinucleotides methylated/total number of CpG dinucleotides sequenced × 100.

### Expression Analysis and 5-aza-2-deoxycitidine treatment

Cell lines were maintained in DMEM media (Sigma Aldrich) supplemented with 10% FCS and 2 mM glutamine at 37°, 5% CO_2_. Breast cancer cell lines HCC1806, HCC1419, HCC1937, HCC1395, MCF7, HTB19, T47D and MDA-MB-231 were treated with 5 μM 5-aza-2'-deoxycitidine (5-aza-dC). Treatment was carried out over 5 days with daily media changes and addition of fresh 5-aza-dC. RNA bee (AMS biotechnology) was used to extract RNA from 5-aza-dC treated and non-treated cells. Control breast total RNA was bought from AMS Biotechnology. cDNA was synthesised from 1 μg total RNA using SuperScript III (Invitrogen) and random hexamer primers. Touchdown PCR was used for expression analysis (primer sequences are shown in additional file 6 for the final 5 positive genes). GAPDH analysis was carried out concurrently for each gene using primers and PCR conditions described previously [35]. For each gene we used 2 methylated and one unmethylated cell line for expression analysis.

### Real-time RT-PCR

Quantitative real-time RT-PCR was performed as described previously [36]. Briefly, cDNA was made from total RNA extracted from frozen normal and tumour breast tissues. We quantified transcripts of the *TBP *(TATA box-binding protein) gene as the endogenous RNA control. Each sample was normalized on the basis of *TBP *content. Results, expressed as N-fold differences in target gene expression relative to the *TBP *gene (termed N*target*), were determined by the following formula: N*target *= 2Δ^Ctsample^, where the ΔCt value of the sample was determined by subtracting the average Ct value of the target gene from the average Ct value of the *TBP *gene. The N*target *values of the samples were subsequently normalized such that the mean ratio of the normal breast samples would equal a value of 1. The nucleotide sequences of the primers used for PCR amplification were as follows: (a) *EMILIN2*-U (5'-GCTTGAAGAAAGCCACAGATAATGAA-3') and *EMILIN2*-L (5'-CTGAGGCCCCTCTTTTGGATCTA-3') with a *EMILIN2*-specific product size of 116 bp, (b) *TBP*-U (5'-TGCACAGGAGCCAAGAGTGAA-3') and *TBP*-L (5'-CACATCACAGCTCCCCACCA-3') with a *TBP*-specific product size of 132 bp. PCR was performed using the SYBR Green PCR Core Reagents kit (Perkin-Elmer Applied Biosystems). The thermal cycling conditions comprised an initial denaturation step at 95°C for 10 min and 50 cycles at 95°C for 15 s and 65°C for 1 min. Experiments were performed with duplicates for each data point.

### Statistical analysis

Methylation results were summarised using cross-tabulations. Comparisons of methylation rates of genes within the same sample were analysed using McNemar's tests, and exact P-values were obtained. Comparisons of methylation rates between normal and tumour cells were analysed using chi-squared tests. Associations between methylation status and clinicopathological characteristics were assessed using a two-sample t-test for the continuous variable age and chi-squared tests for categorical variables. All analyses were conducted using Stata Version 10.1 (Stata Corporation: College Station, TX, USA).

## List of Abbreviations

MIRA: methylated-CpG island recovery assay; 5-aza-dC: 5-aza-2'deoxycytidine; COBRA: Combined Bisulphite Restriction Analysis; NSCLC: non-small cell lung carcinoma; CRC: colorectal cancer.

## Competing interests

The authors declare that they have no competing interests.

## Authors' contributions

VKH did the experiments involving the breast cancer data (bisulphite modification, COBRA, sequencing, expression and bioinformatics analysis) and analysis of lung, colorectal and prostate cancer cell lines. LBH and RLW carried out analysis in colorectal tumours. T Dansranjavin and RD carried out analysis in prostate tumours. AD did bioinformatics analysis on the MIRA data. IB, DE and CL provided breast tumour DNA samples and NHMEC. IB and SV carried out real-time RT-PCR. ST and GPP performed the MIRA assay and CpG island array hybridisation. T Dobbins did statistical analysis. DG did tissue culture. JM provided breast tumour cell lines and N/T paired lung DNA samples. ERM contributed to the concept of the study and statistical analysis. FL conceived the studies, oversaw the experimental work, wrote the manuscript and established all the collaborations. All authors have read and approved the final manuscript.

## Supplementary Material

Additional file 1 **High Stringency Gene List**. This file shows all the genes which fulfilled the highest stringency criteria, 1 or more promoter probes scoring greater than 10 in 4 or more cell lines. Click here for file

Additional file 2 **Functional Annotation using the DAVID Bioinformatics Resource**. This file shows the DAVID functional annotation table for the 32 selected candidate genes http://david.abcc.ncifcrf.gov/. Click here for file

Additional file 3 **Clinical-pathological features of breast cancer cell lines**. Table shows the clinical-pathological characteristics of the breast cancer cell lines used in this study. Click here for file

Additional file 4 **Clinical-pathological features of breast tumours**. Table shows the clinical-pathological characteristics of the breast tumours used in this study. Click here for file

Additional file 5 **Methylation primers**. CoBRA sequences and annealing temperatures are shown for *DBC1*, *CIDE-A*, *EMILIN2*, *FBLN2 *and *SALL1*. Click here for file

Additional file 6 **Expression primers**. Expression primer sequences are shown for *DBC1*, *CIDE-A*, *EMILIN2*, *FBLN2 *and *SALL1*. Click here for file
