# Supplementary material for: Identification of 5 novel genes methylated in breast and other epithelial cancers
Source: Mol Cancer. 2010 Mar 5;9:51. doi: 10.1186/1476-4598-9-51 (PMC2841122; doi:10.1186/1476-4598-9-51)
Supplement: Additional file 3 — Clinical-pathological features of breast cancer cell lines. Table shows the clinical-pathological characteristics of the breast cancer cell lines used in this study. [file 1476-4598-9-51-S3.DOC]

Sample name cell type Diagnosis Age (y) ER PR

NHMEC(1585T) mammary epithelial no cancer 32 negative negative

NHMEC(3736T) mammary epithelial no cancer no data no data no data

HCC1806 Breast cancer squamous cell carcinoma 60 negative negative

HCC1419 Breast cancer primary ductal carcinoma 42 negative negative

HCC1395 Breast cancer primary ductal carcinoma 43 negative negative

HCC1143 Breast cancer primary ductal carcinoma 52 negative negative

HCC1937 Breast cancer primary ductal carcinoma 23 negative negative

MCF7 Breast cancer adenocarcinoma 69 positive no data

T47D Breast cancer ductal carcinoma 54 positive positive

MDA-MB-231 Breast cancer adenocarcinoma 51 negative negative

HTB19 (BT-20) Breast cancer carcinoma 74 negative no data

ER, estrogen receptor status

PR, progesterone receptor status
