# Supplementary material for: Identification of 5 novel genes methylated in breast and other epithelial cancers
Source: Mol Cancer. 2010 Mar 5;9:51. doi: 10.1186/1476-4598-9-51 (PMC2841122; doi:10.1186/1476-4598-9-51)
Supplement: Additional file 4 — Clinical-pathological features of breast tumours. Table shows the clinical-pathological characteristics of the breast tumours used in this study. [file 1476-4598-9-51-S4.DOC]

Number of patients

Age

≤ 60 27

> 60 13

SBR histological grade

I+II 23

III 15

Lymph node status

≤ 3 32

> 3 8

Macroscopic tumour size

≤ 30 mm 26

> 30 mm 13

PR status

Negative 18

Positive 22

ER status

Negative 17

Positive 23

Menopausal

Pre 15

Post 25

Relapse

Yes 17

No 23
