# Supplementary material for: Identification of 5 novel genes methylated in breast and other epithelial cancers
Source: Mol Cancer. 2010 Mar 5;9:51. doi: 10.1186/1476-4598-9-51 (PMC2841122; doi:10.1186/1476-4598-9-51)
Supplement: Additional file 5 — Methylation primers. CoBRA sequences and annealing temperatures are shown for DBC1, CIDE-A, EMILIN2, FBLN2 and SALL1. [file 1476-4598-9-51-S5.PPTX]

## Slide 1
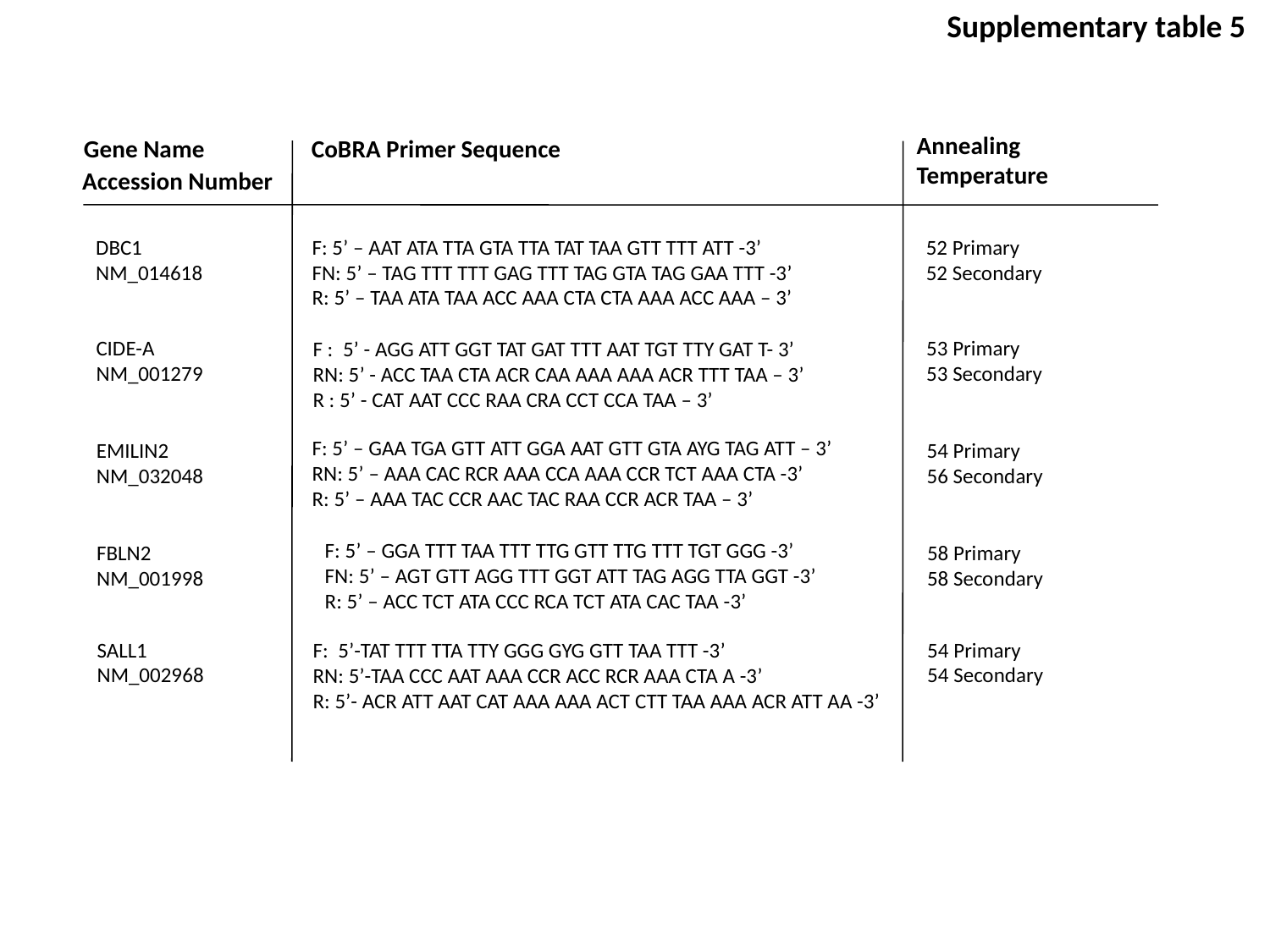

Supplementary table 5
Annealing Temperature
Gene Name
CoBRA Primer Sequence
Accession Number
F: 5’ – AAT ATA TTA GTA TTA TAT TAA GTT TTT ATT -3’
FN: 5’ – TAG TTT TTT GAG TTT TAG GTA TAG GAA TTT -3’
R: 5’ – TAA ATA TAA ACC AAA CTA CTA AAA ACC AAA – 3’
DBC1
NM_014618
52 Primary
52 Secondary
CIDE-A
NM_001279
53 Primary
53 Secondary
F :  5’ - AGG ATT GGT TAT GAT TTT AAT TGT TTY GAT T- 3’
RN: 5’ - ACC TAA CTA ACR CAA AAA AAA ACR TTT TAA – 3’
R : 5’ - CAT AAT CCC RAA CRA CCT CCA TAA – 3’
F: 5’ – GAA TGA GTT ATT GGA AAT GTT GTA AYG TAG ATT – 3’
RN: 5’ – AAA CAC RCR AAA CCA AAA CCR TCT AAA CTA -3’
R: 5’ – AAA TAC CCR AAC TAC RAA CCR ACR TAA – 3’
EMILIN2
NM_032048
54 Primary
56 Secondary
F: 5’ – GGA TTT TAA TTT TTG GTT TTG TTT TGT GGG -3’
FN: 5’ – AGT GTT AGG TTT GGT ATT TAG AGG TTA GGT -3’
R: 5’ – ACC TCT ATA CCC RCA TCT ATA CAC TAA -3’
FBLN2
NM_001998
58 Primary
58 Secondary
SALL1
NM_002968
54 Primary
54 Secondary
F:  5’-TAT TTT TTA TTY GGG GYG GTT TAA TTT -3’
RN: 5’-TAA CCC AAT AAA CCR ACC RCR AAA CTA A -3’
R: 5’- ACR ATT AAT CAT AAA AAA ACT CTT TAA AAA ACR ATT AA -3’
