# Supplementary material for: Identification of 5 novel genes methylated in breast and other epithelial cancers
Source: Mol Cancer. 2010 Mar 5;9:51. doi: 10.1186/1476-4598-9-51 (PMC2841122; doi:10.1186/1476-4598-9-51)
Supplement: Additional file 6 — Expression primers. Expression primer sequences are shown for DBC1, CIDE-A, EMILIN2, FBLN2 and SALL1. [file 1476-4598-9-51-S6.PPTX]

## Slide 1
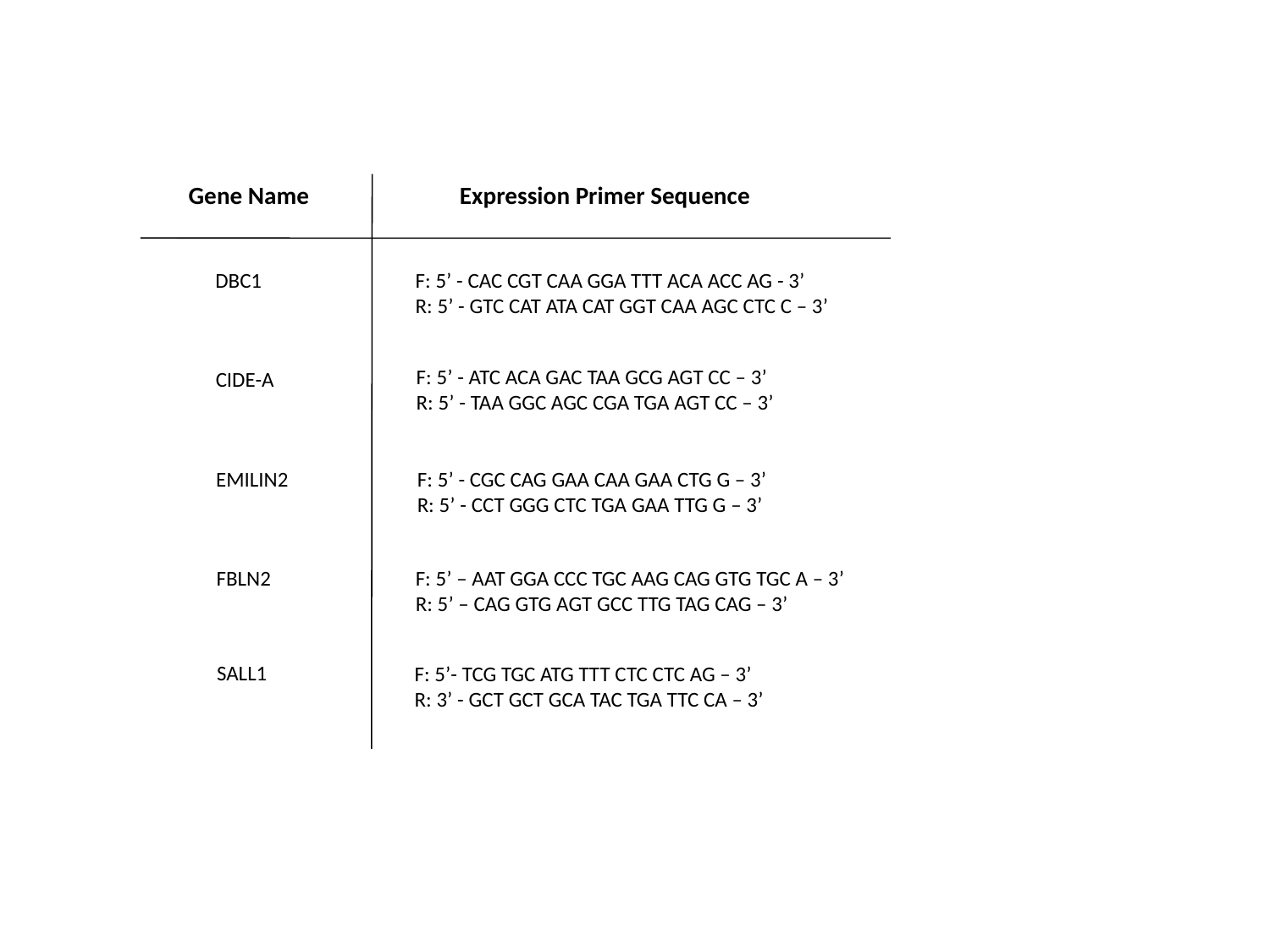

Gene Name
Expression Primer Sequence
F: 5’ - CAC CGT CAA GGA TTT ACA ACC AG - 3’
R: 5’ - GTC CAT ATA CAT GGT CAA AGC CTC C – 3’
DBC1
F: 5’ - ATC ACA GAC TAA GCG AGT CC – 3’
R: 5’ - TAA GGC AGC CGA TGA AGT CC – 3’
CIDE-A
EMILIN2
F: 5’ - CGC CAG GAA CAA GAA CTG G – 3’
R: 5’ - CCT GGG CTC TGA GAA TTG G – 3’
F: 5’ – AAT GGA CCC TGC AAG CAG GTG TGC A – 3’
R: 5’ – CAG GTG AGT GCC TTG TAG CAG – 3’
FBLN2
SALL1
F: 5’- TCG TGC ATG TTT CTC CTC AG – 3’
R: 3’ - GCT GCT GCA TAC TGA TTC CA – 3’
